# Deregulation of the spindle assembly checkpoint is associated with paclitaxel resistance in ovarian cancer

**DOI:** 10.1186/s13048-018-0399-7

**Published:** 2018-04-04

**Authors:** Taryne Chong, Amila Sarac, Cindy Q. Yao, Linda Liao, Nicola Lyttle, Paul C. Boutros, John M. S. Bartlett, Melanie Spears

**Affiliations:** 1 Diagnostic Development, Ontario Institute for Cancer Research, MaRS Centre, 661 University Avenue, Suite 510, Toronto, Ontario M5G 0A3 Canada; 2 Informatics Program, Ontario Institute for Cancer Research, MaRS Centre, 661 University Avenue, Suite 510, Toronto, Ontario M5G 0A3 Canada; 3 Department of Medical Biophysics, University of Toronto, 101 College Street, Room 15-701, Toronto, Ontario M5G 1L7 Canada; 4 Department of Pharmacology and Toxicology, University of Toronto, 1 King’s College Circle, Room 4207, Toronto, Ontario M5S 1A8 Canada; 5 Department of Laboratory Medicine and Pathobiology, University of Toronto, 27 King’s College Circle, Toronto, Ontario M5S 1A1 Canada; 6 Biomarkers and Companion Diagnostics, Edinburgh Cancer Research Centre, Crewe Road South, Edinburgh, EH4 2XR UK

**Keywords:** Spindle assembly checkpoint, Ovarian cancer, Paclitaxel, Mitotic checkpoint serine/threonine kinase (BUB1), centromere protein E (CENPE), Centromere protein F (CENPF), Cyclin B1 (CCNB1)

## Abstract

**Background:**

Ovarian cancer is the leading gynecologic cancer diagnosed in North America and because related symptoms are not disease specific, this often leads to late detection, an advanced disease state, and the need for chemotherapy. Ovarian cancer is frequently sensitive to chemotherapy at diagnosis but rapid development of drug resistance leads to disease progression and ultimately death in the majority of patients.

**Results:**

We have generated paclitaxel resistant ovarian cell lines from their corresponding native cell lines to determine driver mechanisms of drug resistance using gene expression arrays. These paclitaxel resistant ovarian cells demonstrate: (1) Increased IC_50_ for paclitaxel and docetaxel (10 to 75-fold) and cross-resistance to anthracyclines (2) Reduced cell apoptosis in the presence of paclitaxel (3) Gene depletion involving mitotic regulators BUB1 mitotic checkpoint serine/threonine kinase, cyclin BI (CCNB1), centromere protein E (CENPE), and centromere protein F (CENPF), and (4) Functional data validating gene depletion among mitotic regulators.

**Conclusions:**

We have generated model systems to explore drug resistance in ovarian cancer, which have revealed a key pathway related to the spindle assembly checkpoint underlying paclitaxel resistance in ovarian cell lines.

**Electronic supplementary material:**

The online version of this article (10.1186/s13048-018-0399-7) contains supplementary material, which is available to authorized users.

## Background

Each year approximately 158,000 women die of ovarian cancer worldwide [1]. Ovarian cancer is the most common gynecological cancer diagnosed in North America and has one of the lowest survival rates among all cancers [2]. Symptoms are not disease specific and often overlap with other common gastrointestinal and gynecological conditions, which can result in late detection, an advanced disease state, and the need for chemotherapy [2–4]. Roughly 70% of ovarian cancers are sensitive to chemotherapy treatment at diagnosis, but rapid development of drug resistance and the inability to halt metastasis leads to treatment failure and disease progression when detected at later stages [4–6]. Paclitaxel is a frontline therapy used to treat advanced ovarian cancer, and in many instances, paclitaxel is combined with platinum based therapeutic drugs, such as carboplatin to improve overall survival [4, 7]. Paclitaxel inhibits cell replication by stabilizing microtubule assembly, thereby promoting mitotic arrest at the spindle assembly checkpoint [8–10]. Previous evidence points to numerous components of the spindle assembly checkpoint and mitotic regulation playing a major role in several cancers [11, 12]. We have generated isogenic paclitaxel resistant cell lines from their corresponding native cell lines which reflect the 3 most common ovarian histologic subtypes, these include serous, clear cell and endometrioid subtypes [2]. These pre-clinical models were used to observe cytotoxicity, cell cycle modulation and changes in gene expression to examine the mechanisms driving drug resistance. Lastly, through gene expression profiling we have demonstrated disruption of the spindle assembly checkpoint in the paclitaxel resistant cell lines, indicating a potential therapeutic pathway.

## Methods

### Cell lines and cell culture

Human ovarian cancer cell lines TOV21G (representing clear cell ovarian carcinoma) and TOV112D (representing endometrioid adenocarcinoma) were purchased from American Type Culture Collection (Manassas, VA). The human ovarian epithelial-serous cell line COV504 was purchased from Sigma Aldrich (St. Louis, MO). The identity of each cell line was validated prior to use by short tandem repeat genotyping at The Centre for Applied Genomics at The Hospital for Sick Children (Toronto, ON). All cell lines were cultured in Dulbecco’s modified eagles’ medium (DMEM) supplemented with 2 mM glutamine and 10% heat inactivated fetal bovine serum (FBS) from Life Technologies (Carlsbad, CA) and maintained at 37°C in a 5% CO_2_ atmosphere. The following chemotherapeutic drugs: paclitaxel, docetaxel, doxorubicin, epirubicin and carboplatin were purchased from Sigma Aldrich (St. Louis, MO), dissolved in dimethyl sulfoxide (DMSO) from Sigma Aldrich (St. Louis, MO) and supplemented in complete media at increasing concentrations. Cells were exposed to an incremental dose escalation of paclitaxel (2 nM) for approximately 2 passages, up to a final concentration of 25 nM, once paclitaxel resistance was achieved.

### Cell viability and cytotoxicity assays

IC_50_ values were determined using the CCK-8 assay from Dojindo Molecular Technologies (Rockville, MD). Briefly, all cell suspensions were plated in 100 μl per well across a 96-well plate, allowed to grow for 24 hours and incubated at 37°C in a humidified 5% CO_2_ atmosphere. Following a 24 hour incubation, cells were treated with complete DMEM with 10% FBS and supplemented with or without increasing concentrations of drugs (0, 0.3, 1, 3, 10, 30, 100, 1000 or 3000 nM) in DMSO. After 72 hours of exposure to varying drug concentrations, 10 μl of the CCK-8 assay reagent was added to 90 μl DMEM and the cells were incubated for an additional 4 hours at 37°C in a 5% CO_2_ atmosphere. The absorbance of each sample was measured using a microplate reader at 450 nm from BioRad (Hercules, CA). Negative controls were prepared using cell-free complete DMEM containing the CCK-8 reagent.

### Annexin V staining

Cultured cells were treated with or without paclitaxel for 24 hours prior to collection. According to the manufacturer’s protocol from eBioscience (San Diego, CA); cells were washed once in 1X phosphate buffered saline (PBS) and 1X Binding Buffer from eBioscience, then resuspended in 1X Binding Buffer. Following the addition of 5 μl fluorochrome-conjugated Annexin V staining solution to 100 μl cell suspension, cells were incubated in the dark for 15 minutes at room temperature and evaluated for apoptosis on a flow cytometer from Becton Dickinson (San Jose, CA). Negative controls included cells with vehicle (DMSO) stained for Annexin V and cells without staining.

### Cell cycle analysis

Cell cycle distribution was evaluated by using propidium iodide staining and the BD FACSCanto II system (San Jose, CA). Cells were arrested in G1/S phase by employing a double thymidine block (2 mM) from Sigma Aldrich (St. Louis, MO). The following day, the reaction was halted by replacing media with fresh thymidine containing media and cells were collected at varying time points thereafter. Once the cell pellets were collected, they were washed with 1X PBS (pH 7.2) from Life Technologies (Carlsbad, CA) and fixed with ice cold 80% ethanol from Commercial Alcohols (Tiverton, ON). We used 0.1 mg/ml propidium iodide and 2 mg/ml RNase A purchased from Sigma Aldrich (St. Louis, MO) which were added to each sample prior to incubation in the dark for 30 minutes. The cell cycle data were collected using the BD FACSCanto II system and analyzed using FlowJo software (San Jose, CA).

### Microarray sample submission

Illumina Human HT-12-V4 Bead Chips were used for the whole genome microarray analysis by the University Health Network (UHN) Microarray Centre in Toronto, Canada. Total RNA was extracted with the RNeasy Mini kit from Qiagen (Toronto, ON) and used for profiling gene expression changes.

### Gene expression analysis

Summary-level data from GenomeStudio (defaulted to have no normalization or background correction) were loaded into the R statistical environment (v3.0.2) using the lumi package (v2.12.0) from BioConductor [13]. The remaining samples were transformed using variance-stabilizing transformation (VST) and normalized using robust spline normalization. Samples from the same cell lines (native and drug resistant) were pre-processed together to avoid confounding effects from normalizing multiple cell lines together. Following pre-processing, we used general linear-modeling to identify genes that are differentially expressed in drug resistant cell lines relative to native cell line controls. The gene expression levels across all cell lines were determined using a per-gene linear model that assessed both basal levels and drug resistance-induced effects. Coefficients were fitted to terms representing each effect and the standard errors of the coefficients were adjusted using an empirical Bayes moderation of the standard error [14]. To test if each coefficient was statistically different from zero, we applied model-based t-tests, followed by a false discovery rate (FDR) adjustment for multiple testing [15]. Genes were deemed to be significant if their adjusted p-values were less than or equal to 0.05. All statistical analyses were performed using the limma package (v3.16.8) within the R statistical environment (v3.0.2). Genes showing significant differential gene expression levels between the resistant and native samples across all types of cell lines were loaded into the Cytoscape Reactome Functional Interaction (FI) plugin in Cytoscape (v3.0.2). Symbols were loaded as a gene set with the 2013 version of the FI network. FI network was constructed with FI annotations and linker genes. Spectral clustering and Pathway Enrichment were computed for each module using the Reactome FI plugin functions and the pathways exhibiting FDR < 0.05 were considered enriched.

### Immunoblot analysis

Cell lysates were normalized using the BCA Protein Assay Kit by Pierce and equivalent amounts of total protein were separated by electrophoresis on 4-20% BioRad Mini Protean TGX Precast Gels (Hercules, CA). Gels were transferred to nitrocellulose membranes and incubated with rabbit anti-cyclin B1 (1:1000 dilution) from Cell Signaling (Danvers, MA), mouse anti-BubR1 (1:1000 dilution) from BD Transduction Laboratories (San Jose, CA), rabbit anti-CENPE (1:2000 dilution) from Sigma (Oakville, ON), and rabbit anti-CENPF (1:1000 dilution) from Novus Biologicals (Oakville, ON) antibodies. The visualization of blots was performed using the ChemiDoc Imaging System and accompanying Image Lab software from BioRad (Hercules, CA). The membranes were stripped and re-probed for β-actin (1:10,000 dilution) from Proteintech Group (Rosemont, IL) which served as a loading control.

## Results

### Increased resistance to paclitaxel, docetaxel and anthracyclines in human ovarian cancer cells

To study chemotherapeutic resistance in ovarian cancer cells, we established three paclitaxel resistant cell lines by incremental and continuous exposure to paclitaxel, up to a final concentration of 25 nM. We observed marked increases in IC_50_ values for both paclitaxel and docetaxel (up to 75 fold) in all 3 cell lines representing three different histologic subtypes (Table 1). In addition, we observed distinct cross-resistance to anthracyclines, including epirubicin and doxorubicin (typically 3-10 fold) across all 3 cell line pairs (Table 1). Carboplatin is currently a standard treatment option, and we show cross-resistance to carboplatin in the TOV112D cell line, which may indicate improved clinical benefit in the endometrioid ovarian subtype (Table 1).

**Table 1:** The half maximal inhibitory concentration (IC_50_) in human ovarian cancer cells

	Paclitaxel (nM)	Docetaxel (nM)	Epirubicin (nM)	Doxorubicin (nM)	Carboplatin (uM)
TOV21G N	1.31 ± 0.75	0.17 ± 4.43	6.30 ± 1.90	0.02 ± 1.01	17.99 ± 1.27
TOV21G R	44.64 ± 15.41 (34)	10.73 ± 1.28 (63)	20.06 ± 1.48 (3)	9.90 ± 1.72 (495)	0.59 ± 4.64
TOV112D N	1.49 ± 1.40	0.86 ± 1.50	9.60 ± 1.10	11.19 ± 1.11	80.81 ± 1.08
TOV112D R	28.26 ± 1.06 (19)	13.91 ± 1.11 (16)	34.78 ± 1.10 (3.6)	30.16 ± 1.09 (3)	140.0 ± 1.45 (2)
COV504 N	1.91 ± 1.85	0.30 ± 3.37	31.56 ± 1.48	47.58 ± 1.81	103.68 ± 1.14
COV504 R	75.51 ± 27.44 (40)	23.08 ± 1.25 (77)	290.33 ± 2.20 (9)	250.58 ± 2.50 (5)	35.16 ± 1.35

The IC_50_ in both native (N) and drug resistant (R) ovarian cancer cell lines were determined by incremental and continuous exposure to drug. Drug resistance is clearly defined in all subtypes and most evident in the epithelial serous cell line, represented by COV504 for both taxanes and anthracyclines (± standard deviation, average of 3 independent experiments). The resistance factor is shown in parentheses and highlights drug resistance (resistant IC_50_/native IC_50_) for each cell line pair

### Reduced cell apoptosis in paclitaxel resistant human ovarian cancer cells

During exposure of native and resistant cell lines to increasing concentrations of paclitaxel, we demonstrated that paclitaxel resistant cell lines exhibited reduced cell apoptosis relative to their native counterparts, following exposure to 25 and 1000 nM paclitaxel treatment (Table 2). For example, the percentage of apoptotic cells in the native TOV21G ovarian cells was 59% versus 14% in the resistant cells, following the addition of 25 nM paclitaxel. This result was equally marked in the TOV112D ovarian cells where 63% of native cells were apoptotic versus 4% in the resistant cell line. Lastly, a similar trend was observed in the COV504 ovarian cells where 26% of the native cells were apoptotic compared to 10% of the resistant ovarian cells in the presence of 25 nM paclitaxel. The treatment of resistant ovarian cell lines with 25 nM paclitaxel clearly resulted in reduced cell apoptosis across all subtypes observed.

**Table 2:** Percentages of apoptotic ovarian cells following the absence or presence of paclitaxel

	DMSO	Paclitaxel (25nM)	Paclitaxel (1000 nM)
TOV21G N	6.00	58.60	63.00
	(±0.38)	(±4.50)	(±2.80)
TOV21G R	11.20	13.60^1^	56.70
	(±5.50)	(±1.10)	(±30.70)
TOV112D N	1.80	63.20	82.00
	(±0.18)	(±0.50)	(±0.25)
TOV112D R	2.00	3.70^2^	69.00
	(±0.27)	(±0.57)	(±0.87)
COV504 N	7.40	25.70	49.60
	(±4.40)	(±6.70)	(±25.30)
COV504 R	8.40	10.00^3^	29.60
	(±4.40)	(±1.80)	(±6.20)

Annexin V staining was used to determine the early detection of apoptotic cells and after 72 hours, apoptosis is evident in both native (N) and paclitaxel resistant (R) cell lines, in all three subtypes TOV21G, TOV112D and COV504. Both the native and resistant cell lines were exposed to increasing concentrations of paclitaxel; all three paclitaxel resistant cell lines exhibited reduced apoptotic induction when exposed to 25 and 1000 nM paclitaxel treatment (± standard deviation, average of 3 independent experiments). ^1^(*p* < 0.01) ^2^(*p* < 0.04) ^3^(*p* < 0.05) versus corresponding native groups

### Paclitaxel resistant human ovarian cells overcome G2/M arrest

**Fig. 1:**
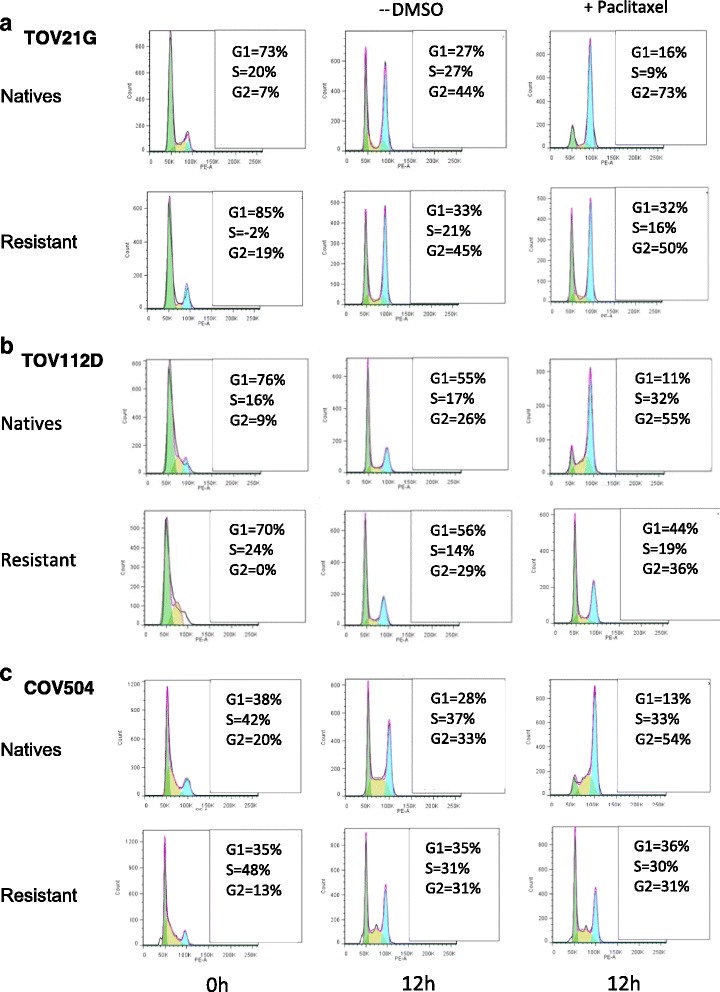
Cell cycle distributions in native and paclitaxel resistant cell lines (**a**) TOV21G, (**b**) TOV112D and (**c**) COV504. Using a double thymidine block, native and paclitaxel resistant cells were synchronized and incubated in the presence of DMSO or 25 nM paclitaxel. Cells were collected at 12 hours and the cell cycle distributions within the cell population were analysed by flow cytometry. The cellular response of 3 cell lines was consistent whereby the resistant ovarian cancer cells treated with paclitaxel were able to overcome paclitaxel induced G2/M arrest and progressed through the cell cycle. The percentages shown represent a single experiment; 3 independent experiments were conducted for each cell line

We monitored cell cycle progression in both the native and resistant ovarian cell lines (±) 25 nM paclitaxel and we found that the resistant cell lines were able to overcome paclitaxel induced G2/M arrest and progress through the cell cycle. At 12 hours, paclitaxel treatment of the native TOV21G, TOV112D and COV504 cells caused a G2/M block and a failure to progress to the G0/G1 phase (Fig. 1). Specifically, the G2/M population of TOV112D native cells increased considerably from 26% to 55% upon exposure to paclitaxel, indicating mitotic arrest in G2/M, compared with a minimal change of 29% to 36% in the TOV112D resistant cells. The increase in cell accumulation at the G2/M phase was accompanied by a decrease of cell population in the G1 phase for the native cells. Similar results were observed in the COV504 resistant cell line, whereby the G1 population changed minimally from 35% to 36% following paclitaxel treatment. These results verify that paclitaxel inhibits cell growth by inducing a block at G2/M phase in several subtypes of ovarian cancer cells, however this effect is more apparent in the native cell lines rather than the resistant cell lines, and overcoming G2/M arrest is a potential process linked to paclitaxel resistance.

### Gene expression analysis in paclitaxel resistant human ovarian cancer cells

**Fig. 2:**
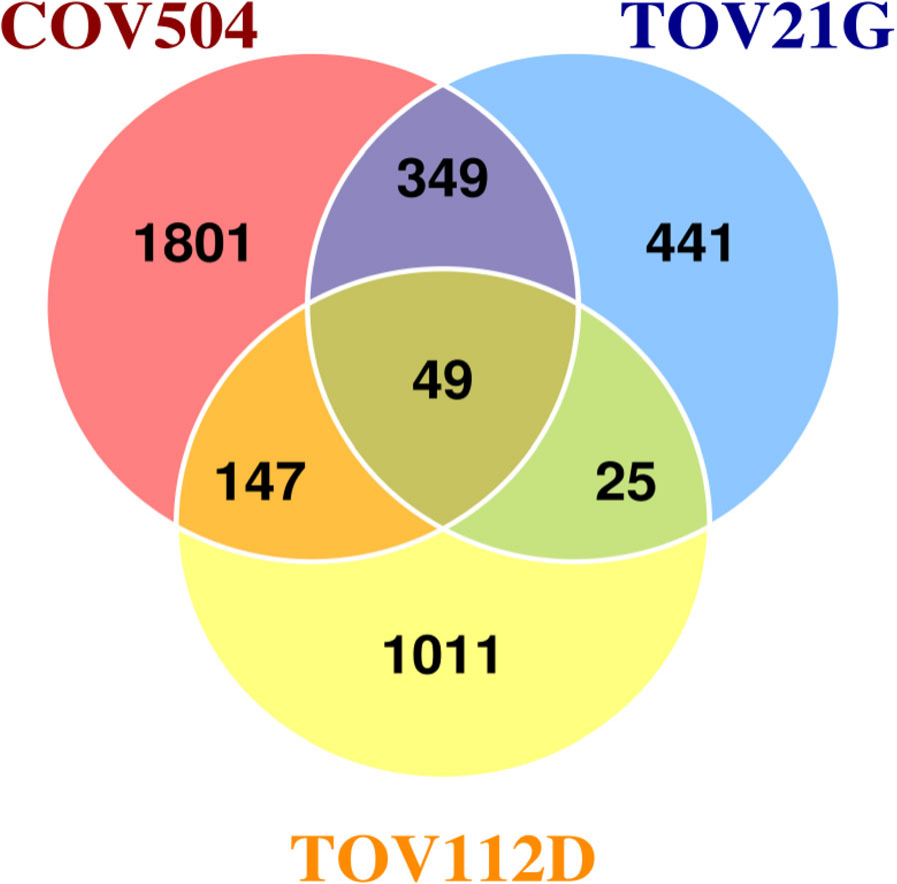
Venn diagram showing overlapping genes between the paclitaxel resistant cell lines. Paclitaxel resistant cell lines TOV21G, TOV112D and COV504 were differentially expressed across multiple histologic subtypes (q ≤ 0.05). The changes in mRNA abundance include an overlap of 49 significant genes highlighting both enrichment and depletion of genes across human ovarian cell lines

The effect of paclitaxel resistance across several subtypes was examined and a number of statistically significant alterations in gene expression levels (q ≤ 0.05) were observed between the native and paclitaxel resistant cells (Fig. 2). These changes in gene expression include 49 deregulated genes across the ovarian histologic subtypes. Among the differentially expressed genes were a number of key regulators that maintain the mitotic spindle checkpoint. Specifically, we determined that 21 genes were found depleted when compared to the native cell lines (Additional file 1: Table S1), and many of these genes were associated with cell cycle regulation and the mitotic checkpoint, and include the following: Aurora kinase A (AURKA), abnormal spindle microtubule assembly (ASPM), BUB1, CCNB1, CENPE, and CENPF and NIMA-related kinase 2 (NEK2). Alternatively, gene enrichment patterns were observed in several functions controlling structural scaffolding and development and apoptotic control; these genes include BCL2/adenovirus E1B 19 kDa interacting protein 3 (BNIP3), sprouty homolog 2 (SPRY2), and the WW domain binding protein 5 (WBP5). Overall, several genes governing G2/M transition were found depleted and to ultimately affect mitotic function, corresponding to acquired paclitaxel resistance in ovarian cell lines.

### Pathway analysis in paclitaxel resistant human ovarian cancer cells

**Fig. 3:**
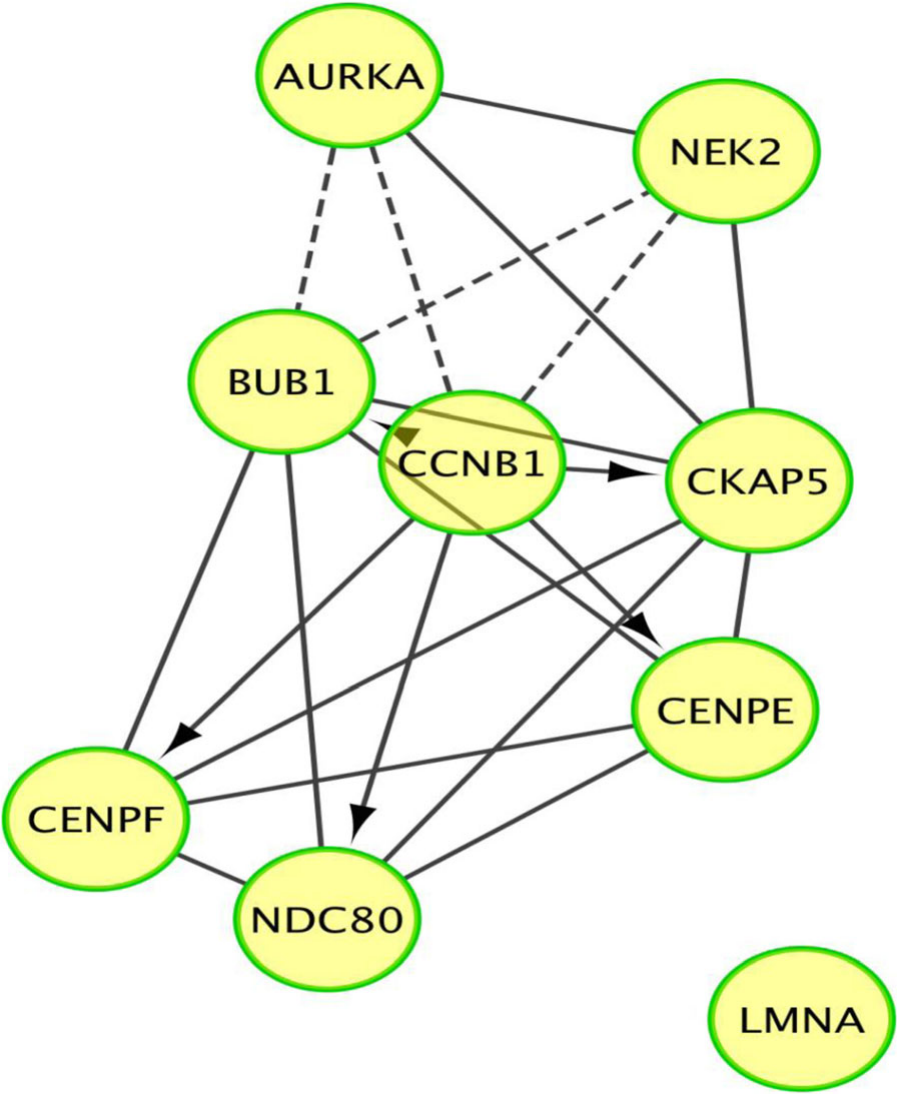
Pathway analysis illustrating differential gene expression and association between gene nodes. The gene nodes display gene depletion (q ≤ 0.05) and include the following candidate genes: BUB1, CCNB1, CENPE and CENPF. The arrows with blunt ends denote negative regulation and sets with overlapping content are connected by a line of long dashes. The arrows represent positive regulation

We explored pathway analysis and observed several gene alterations between resistant cells and their respective native cell lines (q ≤ 0.05) (Fig. 3). The majority of gene nodes display gene depletion and include the following candidate genes: AURKA, ASPM, NEK2, BUB1, CCNB1, CENPE and CENPF. Reactome network analysis revealed significant pathways and genes associated with mitotic regulation, and these include: (1) mitotic pro metaphase (2) mitotic metaphase and anaphase (3) mitotic G2-G2/M phase and (4) APC/C-mediated degradation of cell cycle proteins (Table 3). An additional pathway linked to the spindle checkpoint control involves ubiquitin conjugating enzyme E2C (UBE2C) which was found depleted across the ovarian subtypes. Cell cycle progression was shown to be modified by UBE2C which can affect the degradation of cyclin B1 required for mitotic exit [16].

**Table 3:** Reactome network analysis revealing significant association with mitotic regulation

Associated Pathway	Genes	FDR
mitotic pro metaphase	CCNB1, CKAP5, BUB1, CENPF, CENPE, NDC80	FDR ≤ 3.33 x 10^-4^
mitotic metaphase and anaphase	CKAP5, LMNA, BUB1, CENPF, CENPE, NDC80, UBE2C, PSME1	FDR ≤ 1.00 x 10^-3^
mitotic G2-G2/M phase	CCNB1, CKAP5, NEK2, CENPF, AURKA	FDR ≤ 1.00 x 10^-3^
APC/C-mediated degradation of cell cycle proteins	AURKA, UBE2C, CCNB1, PSME1	FDR ≤ 4.80 x 10^-3^

Reactome network analysis revealed pathways associated with mitotic regulation. These networks predominantly involve spindle assembly checkpoint, centromere-kinetochore complex and cell cycle regulation

### Validation of reduced protein expression in paclitaxel resistant human ovarian cancer cells

**Fig. 4:**
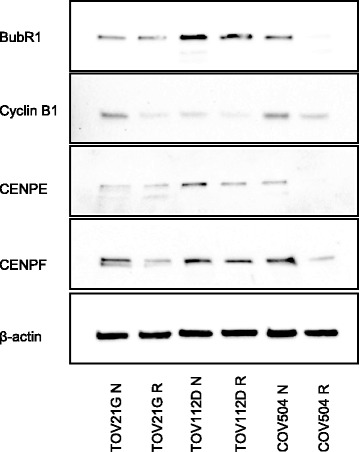
The effect of paclitaxel resistance on key regulators of the mitotic spindle checkpoint. Immunoblot analysis of cell cycle proteins BubR1, cyclin B1, CENPE and CENPF isolated from native and resistant cell lines. Individual lanes contained 20-50 μg of total protein; each gel was normalized. The proteins were resolved on 4-20% Mini Protean TGX Precast Gels, transferred to nitrocellulose membranes and probed using antibodies specific for BubR1, cyclin B1, CENPE and CENPF proteins. β-actin served as a loading control. The immunoblots represent a single experiment; 3 independent experiments were conducted

Immunoblotting of ovarian cancer cells revealed a decrease in spindle assembly checkpoint proteins in the paclitaxel resistant cells (Fig. 4). Specifically, there was a marked decrease in both BUB1-related protein (BubR1) and cyclin B1 protein expression in the paclitaxel resistant cells versus the corresponding natives. Our results support previous work illustrating lower expression of BubR1 and cyclin B1 in paclitaxel resistant ovarian cells OVCAR-3 and SKOV-3 versus their corresponding native cells, thus validating our functional data [8]. Furthermore, a significant decrease in CENPE and CENPF protein expression was observed in the paclitaxel resistant cells versus the corresponding native cells (Fig. 4).

### Clinical tumour samples and kaplan-meier survival curve analysis

**Fig. 5:**
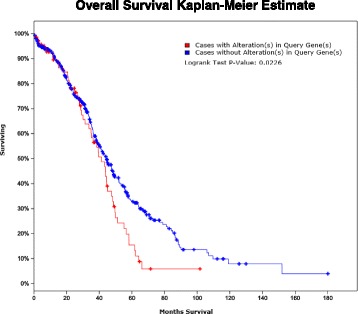
Kaplan-Meier survival curve analysis in primary serous ovarian tumours. Kaplan-Meier survival analysis comparing the mRNA status with overall survival in 489 primary serous ovarian tumour samples. Z-Scores of mRNA expression of candidate genes were downloaded using cBioPortal. Survival status was significantly poorer in patients with altered expression of all 4 candidate genes (*p* < 0.02)

The Cancer Genome Atlas (TCGA) is a large scale database containing human cancer genomics data [17, 18]. We have analyzed our candidate genes using the TCGA clinical database and web based tool cBio Cancer Genomics Portal (http://www.cbioportal.org/), which was developed for mining cancer genome sequencing data [17, 18]. We have evaluated the mRNA expression of primary serous ovarian tumour samples via Agilent microarray to validate the impact of candidate genes BUB1, CCNB1, CENPE and CENPF on overall survival. Using Kaplan-Meier survival curve analysis, we have found that overall survival of 489 patients was significantly less (*p* < 0.02) when the candidate genes were deregulated (Fig. 5). Moreover, patient prognosis with altered expression was significantly poorer than those with unaltered mRNA expression.

## Discussion

Our study revealed gene depletion across a number of molecular components involved in the spindle assembly checkpoint and mitotic regulation, including BUB1, CCNB1, CENPE and CENPF in paclitaxel resistant ovarian cancer cell lines. We found molecular pathways involving mitotic regulation, mitotic pro metaphase, mitotic anaphase and mitotic G2-G2/M phase showing significant depletion. Using the TCGA clinical database, we have shown that altered expression of candidate genes in patients with serous ovarian cancer contributes to significantly poorer survival status in patients. Taken together, we have found evidence of a deregulated spindle assembly checkpoint with significant alteration in mitotic regulators linked to the acquisition of paclitaxel resistance across ovarian subtypes. Several chemotherapeutic agents, such as paclitaxel, target the spindle assembly checkpoint which affects mitotic progression and arrest, thus emphasizing the importance of the mitotic spindle and associated genes as a therapeutic target [19–21].

Cyclin B1 and cyclin dependent kinases (cdks) are associated with mitosis and cell cycle regulation [8, 22]. Our gene expression data reveal significant depletion of cyclin B1 across all three resistant ovarian subtypes. Our findings are consistent with recent data showing reduced cyclin B1 protein expression in two additional paclitaxel resistant ovarian cell lines, OVCAR-3 and SKOV-3 versus their respective parental cell lines [8, 19]. Furthermore, a functional decrease in cyclin B1 protein expression was observed in conjunction with reduced CDK1 (cdc2) expression in human ovarian cisplatin resistant A2780 cells [22]. Since cyclin B1 is a marker specific for the G2/M phase, the decrease in cyclin B1 is associated with the deregulation and weakening of the G2/M phase of the cell cycle [19]. Therefore, a functional loss of cyclin B1 can be attributed to the destabilization of the spindle checkpoint, thus emphasizing the role of cyclin B1 on cell cycle dynamics and the regulatory mechanisms associated with paclitaxel resistance.

Cell cycle progression is tightly orchestrated and many of the spindle assembly checkpoint genes associated with regulation, including BUB1 and BUBR1 are conserved throughout evolution [19, 23]. BUB1 is a molecular component of the spindle checkpoint and required for proper checkpoint signalling and deletions of BUB1 or BUBR1 were detected in several cancers [12, 24]. Previously, mitotic checkpoint defects and the inactivation of the BUB1 gene have been implicated in human colorectal cancer cells [12, 25]. These colorectal cell lines with loss of altered checkpoint function were also found to show chromosomal instability [12]. Consistent with this finding, one study examining a subset of human colorectal tumours revealed a statistically significant association of low BUB1 and BUBR1 mRNA expression with increased metastasis, and a higher recurrence rate [26]. A corresponding observation found BubR1 protein expression was considerably reduced in polyploid cells, and in 31.3% (21/67) of the human colon adenocarcinoma examined [27]. Collectively, these results illustrate that deregulation of the spindle assembly checkpoint is associated with the lack of BUB1 gene function and this deficiency is associated with paclitaxel resistance.

Kinetochore motor proteins CENPE and CENPF are required for proper microtubule attachment and regulation of the spindle checkpoint [23, 28]. Our results reveal significant depletion of CENPE and CENPF mRNA levels across the ovarian subtypes when compared to native cells. In support of our findings, recent evidence shows CENPF siRNA knockdown resulting in cisplatin resistance in a variant small cell lung carcinoma and a non-small cell lung adenocarcinoma [29]. Similar observations reveal depletion of CENPE resulting in unstable kinetochore-microtubule capture and chromosomal instability [30]. Furthermore, evidence illustrated reduced CENPE mRNA expression found in a human hepatocellular carcinoma (HepG2) versus a normal liver cell line (LO2); the results were further validated using western blot analysis and quantitative real-time PCR [31]. In vitro immunofluorescence staining demonstrated that CENPE is co-localized with BUBR1 at kinetochores [28]. The direct interaction between the centromere-kinetochore complex and BUBR1, along with spindle microtubules is believed to modulate mitotic checkpoint signaling events and thus highlights the interaction of kinetochores, spindle checkpoint control and mitotic progression [24].

## Conclusions

We have generated model systems to explore drug resistance in ovarian cancer, which reveal a defective spindle assembly checkpoint associated with acquired paclitaxel resistance. Our results demonstrate that depletion of spindle checkpoint related genes BUB1, CCNB1, CENPE and CENPF during paclitaxel resistance correlates with significant disruption to the cell cycle, which can lead to the suppression of paclitaxel induced cell death. Specifically, multiple cancers exhibit gene down regulation involving CCNB1 and BUB1, highlighting the relationship between a disrupted spindle assembly checkpoint and tumour formation. Since paclitaxel is an effective microtubule drug and exerts apoptotic effect through the regulation of the spindle assembly checkpoint, restoration of gene function to spindle checkpoint related genes would seem an effective target for minimizing cancer progression.

## Additional file

Additional file 1: **Table S1.** List of significant genes illustrating enrichment and depletion of genes across 3 ovarian cancer cell lines. (XLS 38 kb)

## Abbreviations

ASPM: Asp abnormal spindle homolog, microcephaly associated (Drosophila)
AURKA: Aurora kinase A
BCA: Bicinchoninic acid assay
BNIP3: BCL2/adenovirus E1B 19 kDa interacting protein 3
BUB1: Mitotic checkpoint serine/threonine kinase
BUBR1: BUB1 related protein
CCNB1: Cyclin B1
CDK: Cyclin dependent kinase
CENPE: Centromere protein E
CENPF: Centromere protein F
CKAP5: Cytoskeleton associated protein 5
LMNA: Lamin A/C
NDC80: Kinetochore complex component
NEK2: NIMA-related kinase 2
PSME1: Proteasome activator subunit 1
SPRY2: Sprouty homolog 2
UBE2C: Ubiquitin conjugating enzyme E2C
WBP5: WW domain binding protein 5
